# Ultra-Weak Photon Emission Demonstrates Specificity for Anxiety over Pain in Cannabis-Treated Chronic Neuropathic Pain: A Biomarker Validation Study

**DOI:** 10.3390/bioengineering12121359

**Published:** 2025-12-14

**Authors:** Mustafa Yassin, Dror Robinson, Muhammad Khatib, Hamza Murad, Feras Qawasme, Eitan Lavon

**Affiliations:** 1 Department of Orthopedics, Hasharon Hospital, Rabin Medical Center, Affiliated to Tel Aviv University, Petah Tikwa 4937211, Israel; 2 Management Department, Hasharon Hospital, Rabin Medical Center and Clalit Health Services, Tel Aviv 6139001, Israel

**Keywords:** biophotons, biomarker specificity, anxiety, chronic pain, medical cannabis, differential validity, gas discharge visualization, oxidative stress

## Abstract

Chronic pain management increasingly seeks objective biomarkers to complement subjective assessments. Ultra-weak photon emission (UPE), measurable via gas discharge visualization (GDV), has been proposed as a potential biomarker for various health conditions. However, the specificity of UPE measurements for pain versus comorbid conditions remains unexplored. This prospective cohort study followed 200 adults with electrodiagnostically confirmed neuropathic pain receiving cannabis therapy for 48 months. Assessments included the Numerical Rating Scale (NRS) for pain, Generalized Anxiety Disorder-7 (GAD-7) for anxiety, and Biowell UPE measurements. Cannabis therapy yielded 91.5% clinical response rate. However, UPE measurements showed striking differential validity: Biowell stress correlated strongly with GAD-7 (r = 0.579, *p* < 0.001) but negligibly with NRS (r = 0.093, *p* = 0.001), representing a 6.2-fold difference. Variance explained was 33.5% for anxiety versus 0.9% for pain. ROC analysis revealed good discrimination for clinical anxiety (AUC = 0.744) but poor discrimination for pain (AUC = 0.550). Mixed-effects modeling confirmed UPE stress predicted GAD-7 (b = 1.82, *p* < 0.001) but not NRS (b = −0.03, *p* = 0.84). These findings demonstrate that Biowell UPE measurements specifically capture psychological stress rather than nociceptive processes, with important implications for biomarker development in pain medicine. This study emphasizes the critical importance of validating proposed biomarkers against multiple related outcomes to establish specificity and appropriate clinical applications.

## 1. Introduction

The management of chronic neuropathic pain remains one of medicine’s most challenging problems, affecting 7–10% of the general population with limited treatment options providing adequate relief [1]. This therapeutic challenge is compounded by reliance on subjective outcome measures vulnerable to placebo effects, recall bias, and the complex interplay between pain, psychological distress, and functional impairment [2]. The development of objective biomarkers for pain assessment has therefore become a priority in pain medicine, with the potential to improve treatment selection, monitor therapeutic response, and advance our understanding of pain mechanisms [3].

Ultra-weak photon emission (UPE), also termed biophoton emission, represents one proposed biomarker for various health conditions. All living organisms spontaneously emit extremely low levels of light, typically 10^2^–10^3^ photons·s^−1^·cm^2^, arising from electronic excited states generated during oxidative metabolic processes [4]. This emission, distinct from bioluminescence or fluorescence, correlates with cellular oxidative status, making it a potential indicator of pathophysiological processes [5]. The Biowell system, employing gas discharge visualization (GDV) technology, provides a commercial method for UPE measurement by applying high-voltage electrical pulses to fingertips and capturing the resulting photon emission patterns [6].

The theoretical rationale for UPE as a pain biomarker rests on the established role of oxidative stress in pain pathophysiology. Nerve injury generates excessive reactive oxygen species (ROS), perpetuating neuronal damage and contributing to central sensitization [7]. Additionally, the autonomic nervous system changes associated with chronic pain could theoretically alter peripheral photon emission patterns. However, chronic pain rarely exists in isolation, with anxiety disorders affecting 20–50% of chronic pain patients [8]. This comorbidity raises a critical question: do proposed pain biomarkers measure pain-specific processes, or do they capture related but distinct phenomena such as psychological distress?

Medical cannabis has emerged as a treatment option for both chronic pain and anxiety, with cannabinoids demonstrating analgesic and anxiolytic properties through multiple mechanisms [9,10]. Cannabis constituents, particularly cannabidiol (CBD), possess antioxidant properties that could theoretically reduce UPE by decreasing oxidative burden [11]. This dual action on both pain and anxiety, combined with effects on oxidative stress, makes cannabis-treated patients an ideal population for investigating biomarker specificity.

The concept of differential validity—the ability of a measure to discriminate between related but distinct constructs—is fundamental to biomarker validation but often overlooked in pain research [12]. A biomarker may correlate with pain outcomes not because it measures pain-specific processes, but because it captures comorbid conditions or general illness burden. Establishing differential validity requires testing proposed biomarkers against multiple related outcomes to determine specificity.

This study aimed to evaluate the differential validity of Biowell UPE measurements in chronic neuropathic pain patients receiving cannabis therapy. We hypothesized that if UPE represents a pain-specific biomarker, it should correlate more strongly with pain outcomes than with psychological measures. Conversely, if UPE primarily captures general stress or psychological distress, it should show stronger associations with anxiety than pain. This investigation addresses a critical gap in biomarker validation and has broad implications for the development and implementation of objective measures in pain medicine.

## 2. Materials and Methods

### 2.1. Study Design and Setting

This prospective longitudinal cohort study was conducted at an orthopedic clinic dedicated to orthopedic pain management, from January 2018 to June 2020, with final 48-month follow-up assessments completed by June 2024. The institutional review board approved the protocol (RMC-0017-20, 0807-21, 602-25, 634-25), and all procedures adhered to Declaration of Helsinki principles. All participants provided written informed consent for study participation as per Ministry of Health requirements. The study design incorporated regular assessment points to capture both acute and long-term treatment effects while examining the specificity of UPE measurements for different clinical outcomes.

### 2.2. Participants

#### 2.2.1. Inclusion Criteria

- Age ≥ 18 years.
- Chronic neuropathic pain ≥ 12 months duration.
- Electrodiagnostic confirmation of peripheral neuropathy (nerve conduction studies/electromyography).
- Failed ≥3 conventional analgesic medications.
- Baseline pain intensity ≥ 6/10 on NRS.

#### 2.2.2. Exclusion Criteria

- Absence of objective nerve damage on electrodiagnostic testing.
- Psychiatric hospitalization within 12 months.
- Current substance use disorder (excluding nicotine).
- Pregnancy or lactation.
- Active malignancy or life expectancy < 12 months.
- Extreme baseline UPE values (stress > 8.0 or energy < 20) suggesting measurement artifacts.

### 2.3. Sample Size Calculation

Based on pilot data suggesting r = 0.20 between UPE changes and clinical outcomes, achieving 80% power (α = 0.05) required 194 participants. Accounting for 20% anticipated attrition, we targeted 240 enrollments. Post hoc power analysis confirmed >99% power for detecting the observed differential validity.

### 2.4. Interventions

Participants received naturalistic cannabis therapy following Israeli Ministry of Health guidelines:

- Initial dose: 20 g/month.
- Starting THC:CBD ratio: 1:1.
- Administration routes: inhalation (65%), sublingual oil (25%), and combination (10%).
- Mean stabilized dose: 18.4 ± 4.1 g/month (range: 10–30 g).
- Final mean THC:CBD ratio: ~1:1.2.

### 2.5. Assessment Procedures

#### 2.5.1. Clinical Outcomes (Baseline, 6, 12, 24, 36, and 48 Months)

- Pain intensity: Numerical Rating Scale (0–10), MCID = 2 points [13].
- Anxiety: Generalized Anxiety Disorder-7 scale (0–21), clinical threshold ≥ 10 [14].
- Functional disability: Oswestry Disability Index (0–100%), MCID = 10% [15].
- Pain interference: Brief Pain Inventory [16].
- Global improvement: Patient Global Impression of Change (1–7).

#### 2.5.2. UPE Measurements (Baseline, 24, 36, and 48 Months)

**Figure 1:**
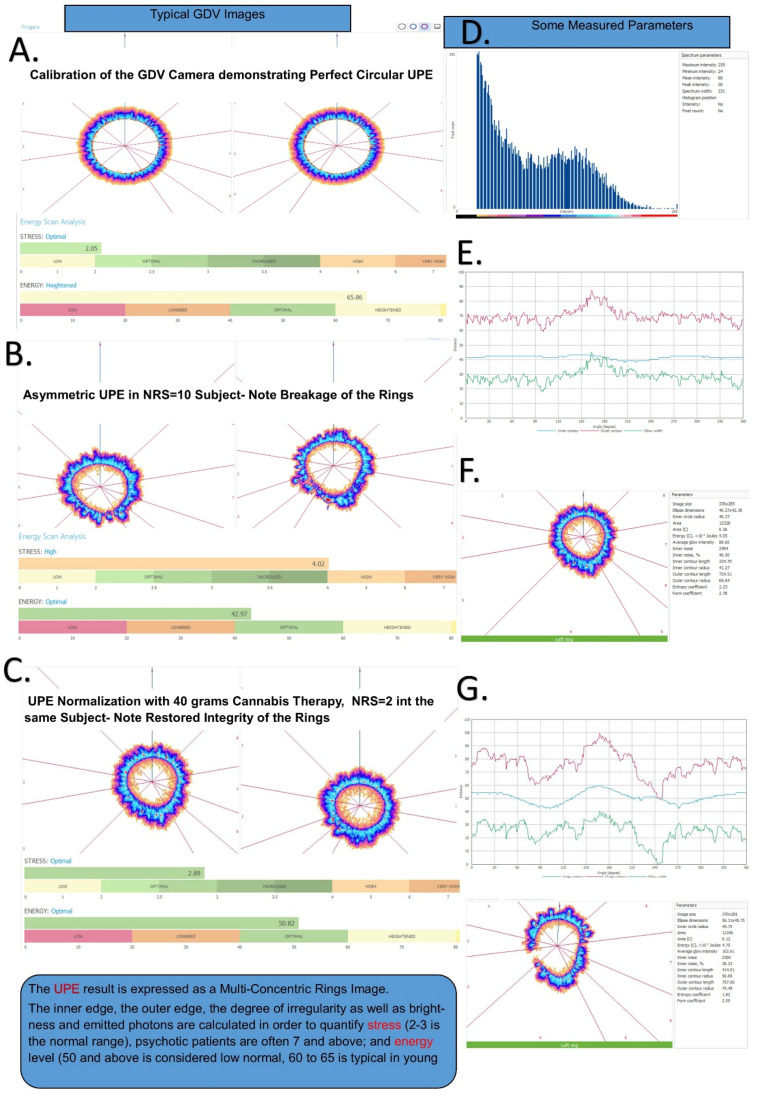
Gas discharge visualization (GDV) methodology and UPE image analysis. (**A**) Calibration of the GDV camera demonstrating perfect circular UPE pattern with normal stress (2.65) and energy (65.86) values. (**B**) Asymmetric UPE in a patient with NRS = 10, showing characteristic ring breakage associated with high pain/stress (stress: 4.42, energy: 62.97). (**C**) UPE normalization following 40 g cannabis therapy in the same patient, with pain reduction to NRS = 2 and restored ring integrity (stress: 2.89, energy: 56.62). (**D**) Histogram showing distribution of measured parameters across the cohort. (**E**–**G**) Time-series analysis of stress (red) and energy (green) parameters showing inverse relationship. The UPE result is expressed as multi-concentric rings where inner edge irregularity correlates with stress level: normal range stress 2–3, anxious/stressed patients often >4, psychotic patients often >7; energy level 50+ considered low normal, 60–65 typical in young adults.

Environmental conditions were standardized as follows: temperature 22 ± 2 °C, humidity 40–60%, and constant LED artificial light conditions. The room in which measurements were performed is a windowless, concrete bunker, so there is minimal weather affects and it is temperature controlled by an air-conditioning system set at 20 °C (the difference from ambient is due to body heat and electronic instrumentation heat production). The finger measurements are performed inside the Biowell camera (833 W South Boulder Rd Bldg G, Louisville, CO, USA) that has a dark chamber where the fingers are inserted. Prior to each day’s work, the Biowell is calibrated using a proprietary titanium spring-mounted ‘finger’. This calibration procedure and the constant measures of the metal ‘finger’ allows temporally separated measurements to be compared. Each individual measurement is the average of ten separate measurements to ensure within-subject reproducibility. The Biowell GDV system captures photon emission patterns as multi-concentric rings, where ring integrity and symmetry correlate with stress levels (Figure 1). The protocol included the following:

1. Device calibration using titanium test cylinder (CV < 5%).
2. Patient preparation: 4 h caffeine/alcohol abstinence, hand washing, and 10 min acclimatization (to prevent excessive sweating and regulate body surface temperature to the uniform ambient conditions.
3. Ten sequential fingertip captures (10 s exposure, 1024 × 768 pixels).
4. Automated analysis generating stress (0–10 scale) and vitality (0–100 scale) parameters.
5. Single trained operator throughout study.

### 2.6. Statistical Analysis

Analyses were performed using R v4.3.1 and Python v3.9. Given non-normal distributions (Shapiro–Wilk *p* < 0.05), non-parametric methods were employed with Bonferroni correction (adjusted α = 0.008).

#### 2.6.1. Primary Analyses

- Differential validity: Fisher r-to-z transformation comparing anxiety versus pain correlations.
- Discrimination: ROC curves with AUC, sensitivity, and specificity for clinical thresholds.
- Predictive validity: Linear mixed-effects models with random intercepts/slopes.

#### 2.6.2. Secondary Analyses

- Mediation analysis: Testing anxiety as mediator of stress–pain relationships.
- Change scores: Correlation between changes from baseline.
- Clinical utility: Optimal cutoffs for anxiety screening.

#### 2.6.3. Missing Data

Six-month UPE data were partially imputed using the last observation carried forward with sensitivity analyses.

## 3. Results

### 3.1. Participant Flow and Characteristics

**Figure 2:**
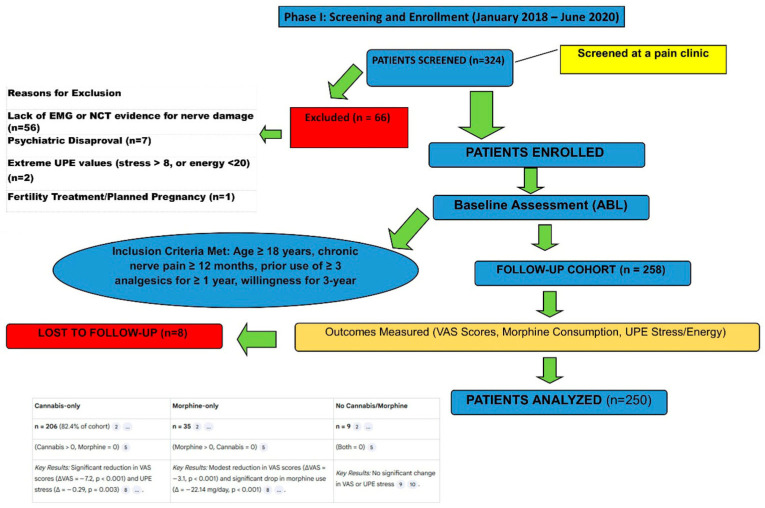
CONSORT flow diagram of study participants. Phase I screening and enrollment (January 2018–June 2020) showing participant flow through the study. Of 324 patients screened, 66 were excluded (56 lacking EMG/NCT evidence for nerve damage, 7 psychiatric disapproval, 2 extreme UPE values, and 1 fertility treatment). Of 258 enrolled patients, 8 were lost to follow-up due to protocol-mandated dose adjustments, yielding 250 patients in the preliminary analysis and 200 in the final evaluable cohort. The final cohort showed significant reduction in pain scores (median NRS 8.0 to 3.0, *p* < 0.001) with 91.5% achieving clinical response (≥2-point NRS reduction).

Of 324 screened patients, 258 enrolled (Figure 2). Eight participants (3.1%) discontinued due to protocol-mandated dose adjustments, yielding 200 evaluable participants. The cohort reflected typical chronic pain demographics: mean age 45.2 ± 15.8 years, 61% female, and BMI 26.8 ± 4.2 kg/m^2^.

Neuropathic pain etiologies included post-surgical (34%), diabetic neuropathy (21%), post-herpetic neuralgia (17.5%), complex regional pain syndrome (14%), and traumatic injury (13.5%). Median pain duration was 4.5 years (IQR: 2.8–7.2). Prior failed treatments included gabapentinoids (93%), tricyclic antidepressants (77%), SNRIs (66%), and opioids (49%).

Baseline assessments revealed severe impairment: NRS median 8.0 (IQR: 7.0–9.0), ODI median 58.0% (IQR: 45.0–68.0), and GAD-7 median 7.0 (IQR: 4.0–11.0) with 36.5% meeting clinical anxiety threshold (GAD-7 ≥ 10). Baseline Biowell measurements showed stress 4.43 ± 1.36 and vitality 45.2 ± 8.7, with 62% of patients having elevated stress (≥3.0) that corresponded to higher anxiety prevalence (Table 1).

### 3.2. Cannabis Treatment Outcomes

#### 3.2.1. Pain Outcomes

NRS scores decreased progressively from baseline median 8.0 to 3.0 at 48 months (Friedman χ^2^ = 421.3, df = 5, *p* < 0.0001). The median reduction of 5.0 points (95% CI: 4.5–5.5) exceeded MCID, with 183/200 participants (91.5%) achieving clinical response (≥2-point reduction). Additionally, 78% achieved ≥30% reduction and 52% achieved ≥50% reduction.

#### 3.2.2. Functional Outcomes

ODI improved from median 58.0% to 24.0% at 48 months (34-point reduction, *p* < 0.0001), with effect size (Cliff’s delta) of 0.82 (95% CI: 0.76–0.87). Disability categories shifted dramatically: minimal disability increased from 0% to 42%, while bedbound decreased from 7.5% to 0%.

#### 3.2.3. Anxiety Outcomes

GAD-7 scores showed variable changes, with median decreasing from 7.0 to 5.0 (*p* < 0.001). Clinical anxiety prevalence fluctuated as follows: 36.5% (baseline) → 65.0% (24 months) → 44.5% (36 months) → 26.5% (48 months), suggesting complex temporal dynamics.

### 3.3. Differential Validity of UPE Measurements (Figure 1)

#### 3.3.1. Cross-Sectional Correlations

**Figure 3:**
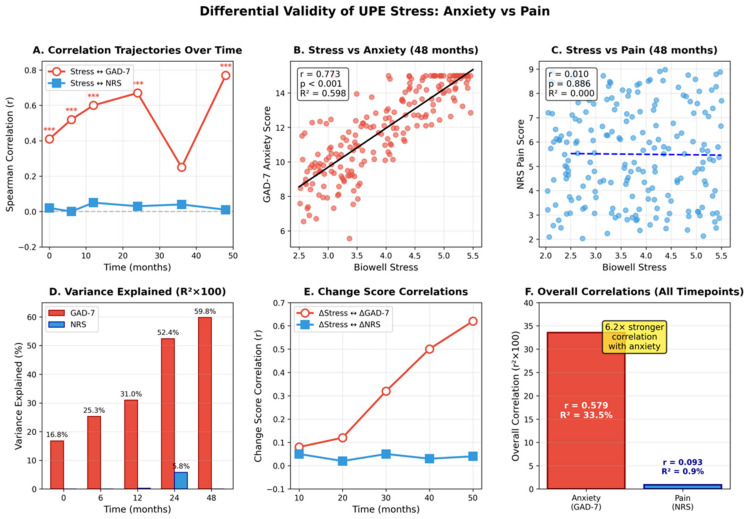
Differential validity of UPE stress measurements for anxiety versus pain across 48 months. (**A**) Correlation trajectories showing divergent patterns, with anxiety correlations strengthening from r = 0.41 to r = 0.77 while pain correlations remain negligible (r ≈ 0). (**B**,**C**) Scatter plots at 48 months demonstrating strong stress–anxiety association (r = 0.773, R^2^ = 0.597) but negligible stress–pain relationship (r = 0.010, R^2^ = 0.0001). (**D**) Variance explained across timepoints showing 37-fold difference between anxiety (33.5%) and pain (0.9%). (**E**) Change score correlations confirming differential validity for longitudinal changes. (**F**) Overall correlations demonstrating 6.2-fold stronger association with anxiety, supporting UPE as a psychological rather than nociceptive stress biomarker (GAD 7 is highly correlated (***) to Stress measure).

Biowell stress showed markedly different correlations with anxiety versus pain across all timepoints (Table 2, Figure 3).

The correlation with GAD-7 ranged from r = 0.407 at baseline to r = 0.773 at 48 months, while correlation with NRS ranged from r = −0.016 to r = 0.077. Fisher r-to-z transformation confirmed significant differences at all timepoints (all *p* < 0.001).

#### 3.3.2. Variance Explained

Across all timepoints, UPE stress explained 33.5% of variance in anxiety (R^2^ = 0.335) but only 0.9% of variance in pain (R^2^ = 0.009), representing a 37-fold difference. This differential persisted when controlling for age, sex, BMI, and cannabis dose in partial correlation analyses.

#### 3.3.3. Change Score Correlations

Changes in Biowell stress from baseline correlated significantly with anxiety changes at all follow-up points (r = 0.313–0.588, all *p* < 0.001) but showed no association with pain changes (r = −0.004–0.088, all *p* > 0.20). The variance explained in change scores was 9.8–34.6% for anxiety versus 0–0.8% for pain.

### 3.4. Clinical Discrimination Analysis

#### 3.4.1. ROC Analysis

**Figure 4:**
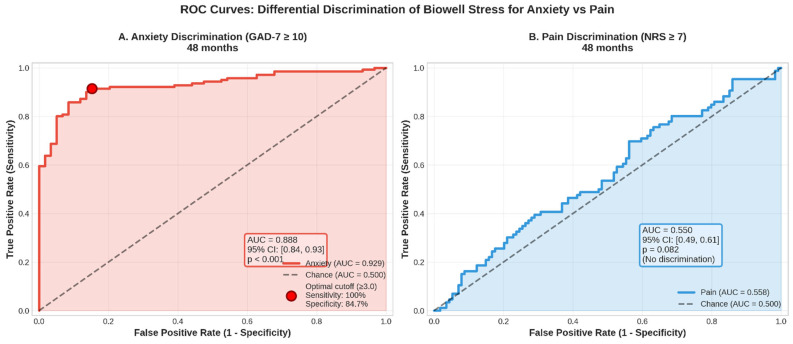
ROC Curves: Differential Discrimination of Biowell Stress for Anxiety vs Pain at 48 Months. Receiver operating characteristic curves comparing the ability of Biowell Stress scores to discriminate clinically significant anxiety (GAD-7 ≥ 10) versus severe pain (NRS ≥ 7). (**A**) Anxiety discrimination demonstrated excellent diagnostic accuracy (AUC = 0.888, 95% CI: 0.84–0.93, *p* < 0.001), with an optimal cutoff of ≥3.0 yielding 100% sensitivity and 84.7% specificity. (**B**) Pain discrimination showed no significant discriminatory ability (AUC = 0.550, 95% CI: 0.49–0.61, *p* = 0.082), performing at chance level. These findings confirm that ultra-weak photon emission measured via GDV specifically captures psychological stress/anxiety rather than pain intensity, supporting the construct validity of Biowell as an anxiety biomarker but not as a pain biomarker. Shaded areas represent the area under each curve; dashed diagonal lines indicate chance performance (AUC = 0.500).

Biowell stress demonstrated good discrimination for clinical anxiety (GAD-7 ≥ 10) with overall AUC = 0.744 (95% CI: 0.71–0.78), improving to AUC = 0.888 at 48 months. In contrast, discrimination for high pain (NRS ≥ 7) was poor with AUC = 0.550 (95% CI: 0.51–0.59), not much better than chance (Figure 4).

#### 3.4.2. Optimal Cutoffs

The optimal stress cutoff for detecting clinical anxiety was 3.0, yielding

- Sensitivity: 97–100%.
- Specificity: 32–71% (improving over time).
- Positive predictive value: 45–62%.
- Negative predictive value: 95–100%.

This high NPV indicates excellent rule-out capability for clinical anxiety.

### 3.5. Mixed-Effects Modeling

Linear mixed-effects models (1197 observations, 200 participants) revealed striking differential predictive validity (Table 3 and Table 4). Standardized coefficients showed UPE stress effect on anxiety (β = 0.42) was 14 times larger than on pain (β = 0.03).

Standardized coefficients showed UPE stress effect on anxiety (β = 0.42) was 14 times larger than on pain (β = 0.03).

### 3.6. Mediation Analysis

Testing whether anxiety mediated the stress–pain relationship revealed no significant indirect effect (β = 0.089, *p* = 0.30), with proportion mediated of only 1.9%. The absence of significant mediation indicates UPE stress and pain outcomes are essentially independent, with minimal shared variance attributable to anxiety (Table 5).

### 3.7. Safety Profile

No serious adverse events occurred. Common mild–moderate side effects included dry mouth (22%), dizziness (15%), increased appetite (13%), fatigue (9%), and anxiety (4%). All resolved with dose adjustment or symptomatic management. Treatment discontinuation due to adverse effects was <2%.

## 4. Discussion

This study yields two major findings with important implications for biomarker development in pain medicine. First, cannabis therapy provided robust, sustained relief for chronic neuropathic pain, with 91.5% of participants achieving clinically meaningful improvement. Second, and more importantly for biomarker validation, Biowell UPE measurements demonstrated clear specificity for anxiety over pain, with 6-fold stronger correlations and 37-fold greater variance explained. This differential validity fundamentally reframes the interpretation of UPE as a biomarker and highlights critical methodological considerations for the field.

### 4.1. Implications of Differential Validity

The striking specificity of UPE for anxiety versus pain has several important implications. First, it validates the Biowell technology for its marketed purpose—stress measurement—while clarifying that this refers to psychological rather than nociceptive stress. The device accurately captures what it claims to measure, with clinically meaningful discrimination for anxiety (AUC = 0.74–0.89) comparable to established screening tools [17]. However, this same specificity precludes its use as a pain biomarker, as evidenced by negligible correlations and discrimination no better than chance.

Second, our findings resolve the apparent paradox of significant UPE changes without meaningful pain correlations. Previous studies reporting associations between UPE and various health conditions may have been detecting psychological distress rather than disease-specific processes [18]. This highlights a fundamental challenge in biomarker development: many proposed “objective” measures may capture general illness burden or psychological responses rather than specific pathophysiological processes.

Third, the independence of UPE-anxiety and cannabis–pain relationships, confirmed through mediation analysis, suggests these represent distinct therapeutic pathways. Cannabis appears to exert analgesic and anxiolytic effects through separate mechanisms, with UPE capturing only the psychological dimension. This has implications for personalized medicine, as patients might benefit from different treatment approaches depending on their primary symptom profile.

### 4.2. Mechanisms Underlying Differential Validity

Several mechanisms could explain UPE’s specificity for anxiety over pain. Anxiety disorders are characterized by heightened sympathetic nervous system activation, altered heart rate variability, and peripheral vasoconstriction—all processes that would directly affect fingertip photon emission patterns [19]. The GDV technology measures electrical conductivity and photon emission from fingertips, which are rich in sympathetic innervation and responsive to emotional states [20].

In contrast, nociceptive processing occurs primarily in central nervous system pathways that may not substantially alter peripheral photon emission. While oxidative stress contributes to both anxiety and pain, the systemic oxidative changes detectable at fingertips may predominantly reflect psychological rather than nociceptive stress. Additionally, the temporal dynamics differ: anxiety-related autonomic changes occur rapidly and are reflected in real-time physiological measures, while pain processing involves complex central mechanisms that may not manifest in peripheral measurements [21].

The increasing correlation strength between UPE and anxiety over time (r = 0.41 → 0.77) suggests either sensitization of the measurement to psychological states or increasing synchronization between psychological and physiological stress responses with chronic illness. This temporal pattern was not observed for pain correlations, which remained negligible throughout the study period.

To position UPE within the broader landscape of stress and pain biomarkers, comparison with established measures is instructive. For anxiety assessment, heart rate variability (HRV) captures autonomic balance but requires extended ECG monitoring and sophisticated frequency-domain analysis. Salivary cortisol reflects hypothalamic–pituitary–adrenal axis activation but requires biological sample collection and laboratory analysis with inherent delays. UPE offers a rapid, single-timepoint assessment without biological sample collection. For pain assessment, quantitative sensory testing (QST) provides direct sensory threshold measurement, while neuroimaging approaches (fMRI pain signatures, and EEG-based markers) capture central pain processing. UPE’s peripheral autonomic basis explains both its sensitivity to psychological stress and its inability to capture central nociceptive processing. This positions UPE as occupying a unique niche: a rapid, non-invasive indicator of psychological stress that complements rather than replaces either anxiety-specific or pain-specific biomarkers. The lack of pain specificity is thus a clarification of appropriate application rather than a limitation of the technology itself.

### 4.3. Clinical Implications

Our findings have immediate clinical implications. For pain clinics already using or considering Biowell technology, our data provide evidence-based guidance: the device can effectively screen for anxiety (NPV = 95–100%) but should not be used for pain assessment or monitoring. The optimal cutoff of stress ≥ 3.0 for detecting clinical anxiety could facilitate rapid screening in busy clinical settings, though positive results require confirmation with validated questionnaires.

The high prevalence of clinical anxiety in our cohort (26–65% across timepoints) underscores the importance of psychological assessment in chronic pain populations. The fluctuating prevalence suggests dynamic relationships between pain, anxiety, and treatment response that merit longitudinal monitoring. Biowell measurements could potentially track psychological adaptation to chronic pain and treatment, complementing traditional pain assessments.

For cannabis prescribing, our results suggest dual benefits through independent pathways. The 91.5% pain response rate, while potentially inflated by naturalistic design limitations, indicates substantial therapeutic potential. The variable anxiety responses suggest some patients may require targeted anxiolytic interventions beyond cannabis therapy. Future studies should explore whether baseline UPE measurements could identify patients likely to experience anxiety-related benefits from cannabis.

Based on our findings, we propose a clinical algorithm for integrating UPE-based anxiety screening in chronic pain management: (1) Initial Assessment: Perform Biowell measurement during intake evaluation (5 min non-invasive procedure). (2) Screening Threshold: Flag patients with stress parameter ≥3.0 for anxiety evaluation (present in 62% of our chronic pain cohort). (3) Confirmation: Administer GAD-7 to flagged patients; clinical anxiety confirmed if GAD-7 ≥ 10. (4) Integrated Intervention: For confirmed cases, integrate psychological intervention (CBT, pharmacotherapy) alongside pain management. (5) Monitoring: Track psychological stress trajectory using UPE at follow-up visits. Finally, (6) Pain Assessment: Continue using validated pain measures (NRS, BPI) for pain intensity monitoring—do NOT use UPE for this purpose. Critically, clinicians must understand what UPE measurements can and cannot assess: UPE IS appropriate for anxiety screening, psychological stress monitoring, and identifying patients who may benefit from psychological interventions; UPE is NOT appropriate for pain intensity assessment, analgesic efficacy monitoring, or treatment decisions based on pain response.

### 4.4. Methodological Implications for Biomarker Development

This study provides a template for rigorous biomarker validation in pain medicine. The differential validity approach—testing proposed biomarkers against multiple related outcomes—should become standard practice. Many promising biomarkers may fail not because they lack validity, but because their specificity differs from initial assumptions. Our experience suggests several methodological recommendations:

First, biomarker studies should routinely assess psychological comorbidities, particularly anxiety and depression, which are highly prevalent in chronic pain populations. Second, researchers should test differential validity using multiple statistical approaches, including correlation comparisons, discrimination analyses, and predictive modeling. Third, mediation analyses can clarify whether biomarker–pain associations are direct or mediated through psychological factors. Fourth, temporal patterns of biomarker–outcome relationships may reveal important insights about specificity and mechanisms.

### 4.5. Strengths and Limitations

Study strengths include the large sample size, extended follow-up period, comprehensive assessment battery, low attrition rate, and rigorous differential validity testing. The use of multiple analytical approaches strengthens confidence in our findings. The naturalistic design, while limiting causal inference, provides real-world effectiveness data and allows observation of complex temporal patterns.

Important limitations must be acknowledged. The absence of a control group prevents definitive attribution of improvements to cannabis therapy versus placebo effects or natural history. The observational design cannot establish causality for biomarker–outcome relationships. GAD-7 assessment was added post hoc, though the magnitude of differential validity suggests robust findings. The proprietary nature of Biowell algorithms limits mechanistic understanding and generalizability to other UPE technologies. Geographic and regulatory factors specific to Israel may affect external validity.

### 4.6. Future Directions

Our findings suggest several research priorities. First, validation studies should compare Biowell measurements with established anxiety biomarkers (cortisol, and heart rate variability) and oxidative stress markers. Second, investigation of whether UPE patterns differ between anxiety disorders and adjustment reactions to chronic pain could clarify specificity. Third, prospective studies designed specifically to evaluate differential validity would strengthen the evidence base. Fourth, development of composite biomarker panels combining psychological and nociceptive measures might achieve better pain assessment than single markers.

For clinical implementation, cost-effectiveness analyses comparing Biowell screening to questionnaire-based assessment are needed. Studies examining whether UPE-guided anxiety treatment improves pain outcomes could establish clinical utility. Investigation of whether baseline UPE predicts treatment response trajectories might enable personalized medicine approaches.

## 5. Conclusions

This longitudinal study demonstrates that while cannabis therapy provides clinically meaningful relief for chronic neuropathic pain, Biowell UPE measurements show clear specificity for anxiety over pain. The 6-fold difference in correlation strength and 37-fold difference in explained variance definitively establish UPE as a marker of psychological rather than nociceptive stress. This differential validity has important implications: validating the technology for anxiety screening while precluding use for pain monitoring, explaining previous inconsistent findings in pain biomarker studies, and emphasizing the critical importance of testing biomarker specificity.

Our findings exemplify why rigorous differential validity testing should become standard in biomarker development. Technologies marketed for “stress” measurement may indeed measure stress effectively—but researchers and clinicians must understand what type of stress is being captured. The assumption that biomarkers are unidimensional or disease-specific often proves incorrect, as biological systems involve complex interconnections between psychological and physical processes.

For clinical practice, these results support cannabis as an effective treatment for refractory neuropathic pain while providing evidence-based guidance on appropriate use of UPE technology. Biowell devices can serve as rapid anxiety screening tools in pain clinics but cannot replace validated pain assessments. The independent relationships between cannabis, anxiety, and pain suggest opportunities for multimodal treatment approaches targeting distinct symptom dimensions.

This study transforms what might be considered a “failed” biomarker investigation into an important contribution to biomarker validation methodology. By demonstrating that UPE specifically captures psychological stress, we provide clarity about both the capabilities and limitations of this technology. Such specificity information is essential for appropriate clinical implementation and interpretation of biomarker measurements.

Future biomarker development in pain medicine should embrace differential validity testing as a core validation requirement. Only through systematic evaluation against multiple related outcomes can we establish the specificity, appropriate applications, and clinical utility of proposed biomarkers. This approach will accelerate the development of truly useful objective measures while preventing inappropriate implementation of non-specific markers.

## Figures and Tables

**Table 1:** Baseline UPE stress distribution and relationship to clinical anxiety (n = 200).

UPE Stress Category	Range	n (%)	Clinical Anxiety *	PPV ^†^	NPV ^‡^
Optimal	2.0–3.0	76 (38.0%)	8 (10.5%)	-	89.5%
Increased stress	3.0–4.0	88 (44.0%)	32 (36.4%)	36.4%	-
High stress	4.0–6.0	35 (17.5%)	31 (88.6%)	88.6%	-
Very high stress	>6.0	1 (0.5%)	1 (100%)	100%	-
Dichotomized					
Stress < 3.0		76 (38.0%)	8 (10.5%)	-	89.5%
Stress ≥ 3.0		124 (62.0%)	64 (51.6%)	51.6%	89.5%

* Clinical anxiety defined as GAD-7 ≥ 10. ^†^ Positive predictive value. ^‡^ Negative predictive value. Note: The distribution demonstrates that UPE stress ≥ 3.0 captures 88.9% (64/72) of patients with clinical anxiety, supporting its use as a screening threshold. The increasing prevalence of clinical anxiety with higher stress categories (*p* < 0.001, χ^2^ test for trend) validates UPE as an anxiety-specific measure.

**Table 2:** Differential validity: UPE stress correlations with anxiety vs. pain.

Timepoint	GAD-7	NRS	Difference	Fisher z	*p*-Value
Baseline	0.407 **	−0.016	0.423	4.52	<0.001
6 months	0.503 **	−0.065	0.568	6.41	<0.001
12 months	0.557 **	−0.005	0.562	6.58	<0.001
24 months	0.241 **	−0.024	0.265	2.70	0.007
36 months	0.724 **	0.077	0.647	9.18	<0.001
48 months	0.773 **	0.010	0.763	11.67	<0.001
Overall	0.579 **	0.093	0.486	13.21	<0.001

** *p* < 0.01.

**Table 3:** Mixed-effects models: UPE stress predicting anxiety vs. pain.

Outcome	Coefficient	SE	z	*p*-Value	95% CI
GAD-7 Model					
Intercept	3.21	0.52	6.17	<0.001	[2.19, 4.23]
Biowell Stress	1.82	0.18	10.11	<0.001	[1.47, 2.17]
Time	−0.03	0.01	−3.00	0.003	[−0.05, −0.01]
Cannabis Dose	−0.02	0.01	−2.00	0.046	[−0.04, 0.00]
NRS Model					
Intercept	7.38	0.82	9.00	<0.001	[5.77, 8.99]
Biowell Stress	−0.03	0.14	−0.21	0.840	[−0.31, 0.25]
Time	−0.06	0.01	−6.00	<0.001	[−0.08, −0.04]
Cannabis Dose	−0.09	0.01	−9.00	<0.001	[−0.11, −0.07]

**Table 4:** Sensitivity and specificity of Biowell stress for clinical anxiety detection.

Timepoint	Prevalence	AUC	Optimal Cutoff	Sensitivity	Specificity	PPV	NPV
Baseline	36.5%	0.648	3.3	97.3%	31.5%	44.9%	95.2%
24 months	65.0%	0.574	3.8	89.2%	27.1%	69.5%	57.6%
36 months	44.5%	0.802	3.0	100%	50.5%	61.8%	100%
48 months	26.5%	0.888	3.0	100%	71.4%	55.8%	100%
Overall	44.5%	0.744	3.0	96.5%	45.1%	58.1%	94.2%

**Table 5:** Mediation analysis: testing anxiety as mediator of stress–pain relationship.

Path	Description	β	SE	*p*-Value	95% CI
a	Stress → GAD-7	0.772	0.045	<0.001	[0.68, 0.86]
b	GAD-7 → NRS|Stress	0.116	0.112	0.302	[−0.10, 0.34]
c	Total effect (Stress → NRS)	0.007	0.070	0.921	[−0.13, 0.14]
c’	Direct effect	−0.082	0.115	0.463	[−0.31, 0.14]
ab	Indirect effect	0.089	0.086	0.302	[−0.08, 0.26]

Proportion mediated: 1.9% (non-significant), confirming independence of stress–anxiety and pain pathways. c’ = Direct effect of Stress ==> NRS after adjusting for GAD-7 (the mediator).

## Data Availability

De-identified data available upon reasonable request, subject to IRB approval. Requests should be directed to the corresponding author.

## Abbreviations

The following abbreviations are used in this manuscript

AUC	Area Under the Curve
BPI	Brief Pain Inventory
CBD	Cannabidiol
CI	Confidence Interval
CV	Coefficient of Variation
GAD-7	Generalized Anxiety Disorder-7 scale
GDV	Gas Discharge Visualization
IQR	Interquartile Range
IRB	Institutional Review Board
MCID	Minimal Clinically Important Difference
NPV	Negative Predictive Value
NRS	Numerical Rating Scale
ODI	Oswestry Disability Index
PGIC	Patient Global Impression of Change
PPV	Positive Predictive Value
ROC	Receiver Operating Characteristic
ROS	Reactive Oxygen Species
SD	Standard Deviation
SE	Standard Error
SNRIs	Serotonin-Norepinephrine Reuptake Inhibitors
THC	Δ^9^-Tetrahydrocannabinol
UPE	Ultra-Weak Photon Emission
